# Effects of Dietary Vegetable Oil Mixtures including Soybean Oil on Intestinal Oxidative Stress in Gilthead Sea Bream (*Sparus aurata*)

**DOI:** 10.3390/ani13061069

**Published:** 2023-03-15

**Authors:** Irene García-Meilán, Ramón Fontanillas, Joaquim Gutiérrez, Encarnación Capilla, Isabel Navarro, Ángeles Gallardo

**Affiliations:** 1 Departament de Biologia Cel·lular, Fisiologia i Immunologia, Facultat de Biologia, Universitat de Barcelona, Av. Diagonal 643, 08028 Barcelona, Spain; 2 Skretting Aquaculture Research Centre (ARC), Sjøhagen 3, 4016 Stavanger, Norway

**Keywords:** lipid peroxidation, superoxide dismutase, catalase, glutathione peroxidase, intestine, soybean oil, fish diet

## Abstract

**Simple Summary:**

Vegetable oil inclusion in fish diets is a common practice, but their effects in the oxidative status at intestinal level, which would affect animal health and welfare, are poorly understood. In the present study, we compared the effects of different dietary treatments containing soybean oil alone or in combination with other vegetable oils in sea bream. Overall, the results revealed that the blend of soybean and linseed oils negatively affects intestinal integrity as it triggered high oxidative stress that could not be counteracted by the high levels of antioxidant enzymes. However, the addition of palm oil to the previous mixture of vegetable oils makes it possible to maintain low oxidative stress, preserving the intestinal health of the animal. In conclusion, this study demonstrates the importance that the mixture of vegetable oils in a diet can have on the intestinal health of sea bream.

**Abstract:**

Fish oil is commonly replaced by vegetable oils in sea bream diets, but little is known about their effects on intestinal health regarding oxidative stress biomarkers. The negative effects of lipid peroxidation on digestive mucosa could have consequences in animal nutrition and welfare. In this study, five isonitrogenous (46%) and isolipidic (22%) diets with 75% of vegetable oils inclusion were evaluated: soybean oil (S) alone or different mixtures containing soybean oil with linseed (SL), linseed and rapeseed (SLR), linseed and palm (SLP), and linseed, rapeseed, and palm (SLRP). Gilthead sea bream juveniles were fed twice a day for 18 weeks. Pyloric caeca and proximal intestine samples were collected 24 h post feeding for lipid peroxidation (LPO), antioxidant enzyme activities (SOD, CAT, GPx, GST, and GR) and gene expression analyses. Pyloric caeca presented larger unhealthy changes in oxidative status than proximal intestine. Although SL-fed fish showed the highest antioxidant activities, they were unable to cope with LPO that in pyloric caeca was 31.4 times higher than in the other groups. Instead, SLP fish presented the best oxidative status, with low LPO levels, antioxidant enzyme activities, and gene expression. In summary, between the vegetable oils dietary mixtures tested, SPL would maintain better intestinal health.

## 1. Introduction

For the sustainability of aquaculture, it is necessary to replace fish oil (FO) with alternative ingredients that maintain the quality, health, and welfare of the fish, while allowing to reduce production costs [1]. This need arises from the high prices of FO due to its use in nutraceutical and agricultural industries because of its health properties [1]. Vegetable oils (VO) are primarily used to replace FO in feed formulation and among the most used vegetable oils, we can find soybean oil, which has 64% of polyunsaturated fatty acids (PUFAs), mainly linoleic acid (C18:3n-6) (57% of the total fatty acids) and rapeseed oil, rich in monounsaturated fatty acids (MUFAs) (57%), mainly oleic acid (C18:1n-9) [2]. Among the VO, there is also linseed oil, very rich in n-3 fatty acids with 75% of PUFA, mainly α-linoleic acid (18:3n-3), moderate content of MUFA (16%) and low in saturated fatty acids (SFA, 9%), but with a high market price, which limits its use in fish feeds [2]. Moreover, we can also find palm oil, rich in SFA and MUFA (50 and 40% of total fatty acids, respectively), its use being limited indeed by its amount of SFA [2]. Therefore, although FO replacement is an unavoidable necessity, an imbalance in the dietary fatty acid profile could negatively affect fish health and welfare, especially in marine species such as gilthead sea bream. In this species, eicosapentaenoic (EPA, 20:5n-3) and docosahexaenoic (DHA, 22:6n-3) acids among others, are essential fatty acids required for proper development and growth [2,3,4,5], and thus, a minimum amount of FO would be necessary in those diets to meet the fish requirements concerning these fatty acids.

It is well known that FO replacement by VO affects directly the gastrointestinal tract by changing the membrane fatty acid composition due to the fast turnover of enterocytes, endangering their primary functions: digestion and absorption [6,7]. The effect of VO is very dependent on dietary factors such as the type of both FO and VO incorporated, the level of FO substitution, the presence and availability of other nutrients (particularly antioxidants) [8] and the duration of feeding. Moreover, it is also influenced by other non-dietary factors such as the fish species, fish size, and life cycle stage, as well as environmental conditions, mainly temperature [9]. However, the use of blends of different VO can reduce or avoid the deleterious effects on growth performance in fish fed diets with less than 7% of fish meal and FO sources [10,11,12].

When delving into the effects at the intestinal level, dietary changes could also provoke morphological alterations that impair intestinal functionality, including paracellular permeability, and epithelial transport functions, specifically, membrane digestive enzymes, amino acids and glucose transporters activities and diffusion rates, thus affecting nutrient utilization [9,13]. In this sense, VO inclusion led to a supranuclear lipid droplet accumulation in Artic charr (*Salvelinus alpinus*) fed a diet that included linseed oil [14] and in rainbow trout (*Oncorhynchus mykiss*) fed a diet including soybean oil [15,16], probably due to a reduction in lipoprotein synthesis as it was described in gilthead sea bream [6,17]. Nevertheless, the total replacement of FO by soybean oil in a diet for Atlantic salmon (*Salmo salar*) did not affect enterocyte lipid accumulation [18]. Moreover, dilated intercellular spaces were found in the intestine of sea bream fed 60 and 80% of soybean oil inclusion reflecting an impaired transit capacity through the lamina propria [17]. Thus, VO inclusion can directly affect the gastrointestinal tract at different levels: membrane composition, structure, integrity, and function. Furthermore, the oxidative status of this organ could also be affected. The formation of reactive oxygen species (ROS) is a natural process caused by cellular metabolism itself and its interaction with the environment. Dietary changes of both lipid content as fatty acid degree of unsaturation and length [19] could modify ROS formation due to an imbalance between production and removal. It is known that an increase in ROS triggers DNA damage, enzyme inactivation, protein oxidation, and lipid peroxidation (LPO) [20]. This could compromise the physical barrier function at intestinal level by causing negative effects on membrane structure, fluidity and permeability [21] and thus, negatively affect fish welfare [22,23,24].

Antioxidants are the main cellular mechanisms to fight ROS and maintain physiological status [20]. Cells have developed enzymatic antioxidant mechanisms against oxidative stress [25], which include superoxide dismutase (SOD), catalase (CAT), glutathione peroxidase (GPx), glutathione S transferase (GST), and glutathione reductase (GR), which is coupled with GPx and GST and recycles oxidised glutathione (GSSH) [20]. Moreover, organisms also have non-enzymatic antioxidants with low molecular weight that directly quench ROS, such as vitamins, carotenes, and glutathione (GSH) among others [20,26].

Specific studies on oxidative status in pyloric caeca and proximal intestine are scarce despite that are multiple external and internal triggers inducting oxidative stress at intestinal level [21,22,27,28,29,30,31,32]. To our knowledge, two studies in this area have been conducted in gilthead sea bream, an important species for Mediterranean aquaculture [22,30]. Castro et al. [22] tested the effect of total replacement of FO by a blend of palm, linseed, and rapeseed oils (30:50:20) in fish fed high-protein diets (50–66% from fishmeal) in whole intestine and they found a protective role of VO against LPO in fish fed diets containing VO versus fish fed a FO diet. Moreover, that study revealed that LPO and antioxidant status were tissue specific, since liver and intestine LPO levels and antioxidant activities showed different profiles. Magalhaes et al. [30] tested diets with the same blend of VO but supplemented with arachidonic acid (ARA, 20:4n-6), EPA and DHA and their results revealed the importance of the ratio n-6/n-3 long-chain (LC)-PUFA content in the proximal intestine oxidative status. In this sense, some other studies have demonstrated that appropriate levels of n-3 LC-PUFA could improve antioxidant capacities against oxidative stress in salmonids [23,33,34].

The present study seeks to investigate if the dietary inclusion of different VO including soybean oil alone or combined and resulting in different n-6/n-3 ratios, could improve the antioxidant status and LPO, both in the pyloric caeca and in the proximal intestine of gilthead sea bream, looking for a healthy impact on fish of the FO substitution by VO while improving the sustainability of the aquaculture sector.

## 2. Materials and Methods

### 2.1. Experimental Diets

Skretting ARC (Norway) formulated and produced five isonitrogenous (46%) and isolipidic (22%) diets (Table 1) in which 25% of included oils was FO and 75% of VO. One diet contained only soybean oil as VO (S diet), in the second diet, soybean and linseed oils were blended (SL diet). In another two other diets, a combination of three VO was used, where soybean and linseed oils were mixed with either rapeseed or palm oils (SLR and SLP diets, respectively) with a similar amount of soybean oil in both of them. Finally, in the last diet, a blend of all VO was included (SLRP diet).

All diets, except diet S, presented high and similar levels of n-3, achieving the optimum levels for this species. Moreover, as soybean oil diminished, the amount of n-6 fatty acids also decreased, producing diets with different n-6/n-3 ratios; however, at the same time, the amount of MUFAs increased due to the inclusion of other VO showing the diets’ different fatty acids profiles (Table 2). All diets contained 88% of vegetable protein from soya concentrate (30%), corn gluten (15%), fava beans (6%), wheat gluten (3.8%), sunflower meal (3%), as well as wheat (7%) and fishmeal (15%).

### 2.2. Fish and Feeding Trial

Three hundred and eight sea bream (81.73 ± 0.36 g) were randomly distributed in a semi-recirculating saltwater system of 14 fiberglass tanks (500 L; 22 fish per tank) and acclimatized for 11 days at the Institut de Recerca i Tecnologia Agroalimentàries (IRTA, La Ràpita, Spain) facilities. The photoperiod followed natural changes (11:24 to 10:29 h of daylight), according to the course of the trial (October-February), and temperature was maintained at 21.9 ± 0.85 °C. During the 18-week trial, fish were fed ad libitum the corresponding diet twice a day (at 8 a.m. and 14 p.m.). Triplicate tanks were used for all experimental conditions, except for the S group where they were in duplicate. The present experiment is part of a more complex study from which data on growth performance have been already published [35].

### 2.3. Sampling Procedures and Samples Preparations

At the end of the growth trial, 3 fish per tank were anaesthetized (MS-222, Sigma, Madrid, Spain), measured, weighed, and sacrificed by severing the spinal cord 24 h post feeding (n = 9 from triplicate groups and n = 6 from duplicate one). The Ethics and Animal Care Committee of the University of Barcelona approved all procedures following the European Union assigned principles and legislation (permit number DAAM 8982).

Samples of pyloric caeca and proximal intestine (PI) were collected, rapidly frozen in liquid nitrogen and maintained at −80 °C. Pyloric caeca and proximal intestine samples were homogenized in buffer solution (Tris-HCl, 50 mM, pH 7.5) using rapid vibration (6500 rpm; 3 × 20 s with three breaks of 20 s; 4 °C) in a Precellys Evolution^®^ Homogenizer combined with Cryolys^®^ as a cooling system (Bertin Technologies, Montigny-le-Bretonneux, France). Next, homogenates were centrifuged for 15 min (2400 rpm; 4 °C; Eppendorf, 5418R) and supernatants were stored at −80 °C until the analyses were performed.

### 2.4. Oxidative Stress Markers Analysis

LPO were determined according to Uchiyama and Mihara [36]. Briefly, homogenized samples were thawed on ice and mixed with HCl 0.024 N and thiobarbituric acid 0.06 M (TBA) solution at pH 7.0. The mixture was heated to 95 °C for 10 min and then kept in ice and darkness for 5 min. Next, 300 µL of cold butanol were added and samples centrifuged for 10 min at 2000 rpm and 4 °C (Eppendorf, 5418R). The concentration of malondialdehyde (MDA) was recorded by fluorescence using a Tecan infinite 200 spectrofluorometer (Tecan Trading AG, Männedorf, Switzerland) with a 515/548 nm (excitation/emission) filter. A calibration curve with MDA in the range of 0–10 µM MDA was used to calculate the MDA concentration and the results were expressed as nmol MDA per mg of protein.

The ferricytochrome C method was used to determine total SOD (EC 1.15.1.1) activity according to Mccord and Fridovich [37], with some modifications, with xanthine/xanthine oxidase as the source of superoxide radicals. Homogenized samples were thawed on ice and then mixed with xanthine oxidase (0.5 IU·mL^−1^) and 200 µL of the reaction buffer (50 mM potassium phosphate buffer at pH 7.8, 0.1 mM EDTA, 0.095 mM cytochrome C, and 0.015 mM xanthine). One unit of activity was defined as the amount of enzyme necessary to produce 50% inhibition of ferricytochrome C reduction rate and normalized by mg of protein. CAT (EC 1.11.1.6) activity was determined according to the method described by Aebi [38], with some modifications. Homogenized samples were thawed on ice and mixed with the reaction buffer containing: 50 mM potassium phosphate buffer at pH 7.0 and, 10 mM H_2_O_2_ freshly added and the disappearance of H_2_O_2_ was measured at 240 nm. For GPx (EC 1.11.1.9) activity determination, samples were reacted with a 50 mM potassium phosphate buffer (pH 7.2), 1.33 mM EDTA, 2.66 mM sodium azide, 40 mM GSH, 4 U·mL^−1^ GR, 2 mM NADPH, and 1 mM H_2_O_2_, and the rate of NADPH oxidation in the coupled reaction with glutathione reductase was determined at 340 nm according to Bell et al. [39]. GST (EC 2.5.1.18) was evaluated as previously described by Habig et al. [40] with some modifications. The assay determined the formation of an adduct between the oxidant agent 1-chloro-2,4-dinitrobenzene (CDNB) and GSH as an increase in DO at 340 nm. The reaction buffer contains: 100 mM potassium phosphate buffer (pH 6.5), 0.1% Triton X-100, 50 mM GSH and 40 mM CNDB. GR (EC 1.6.4.2) activity was determined according to Carlberg and Mannervik [41] by measuring the NADPH consumption rate. Homogenized samples were thawed on ice and reacted with a 0.1 mM potassium phosphate buffer (pH 7.5), EDTA 1 mM, NADPH 0.66 mM, and GSSG 3.25 mM and NADPH oxidation was measured at 340 nm. All substrates, reagents, coenzymes, and purified enzymes were from Sigma and Bio-Rad Laboratories, Inc. All enzymatic analyses were performed at 25 °C ± 0.5 °C using a Tecan M200 spectrophotometer (Tecan Trading AG, Männedorf, Switzerland). CAT, GPx, GR, and GST enzymatic activities are shown as U per mg of protein, where one unit is defined as the amount of enzyme required to transform 1 μmol of the substrate per minute, under the assay conditions.

The protein concentration in homogenates was determined by the Bradford method [42] using bovine serum albumin as a standard.

### 2.5. RNA Extraction and cDNA Synthesis

Total RNA extraction was performed from 30 mg of pyloric caeca or proximal intestine tissue samples in 1 mL TRIzol^®^ reagent solution (Applied Biosystems, Madrid, Spain) following the manufacturer’s instructions. RNA concentration and purity were determined with a Nanodrop 2000 (Thermo Scientific, Alcobendas, Spain). RNA integrity was checked with a 1% agarose gel stained with SYBR-Safe DNA gel stain (Life Technologies, Alcobendas, Spain). To eliminate all genomic DNA, 1 µg of total RNA were treated with DNase I (Invitrogen, Alcobendas, Spain) following the manufacturer’s recommendations before cDNA synthesis. Finally, reverse transcription was performed using the Transcriptor First Strand cDNA synthesis kit (Roche, Sant Cugat del Vallès, Spain) following the manufacturer’s instructions, using anchored-oligo(dT)15 and random hexamer primers.

### 2.6. Real-Time Quantitave-PCR (qPCR)

Gene expression (mRNA) analyses were performed by qPCR in a CFX384 real-time system (Bio-Rad, El Prat de Llobregat, Spain), according to the requirements of the MIQE guidelines [43]. The antioxidant genes examined in pyloric caeca and proximal intestine, all previously validated for gilthead sea bream [44,45,46] were the following: superoxide dismutase 1 and 2, *sod1* and *sod2*, respectively; catalase, *cat*, GPx mitochondrial, *gpx1*, and cytosolic, *gpx4, gr* and *gst*; and three reference genes (beta actin, *β-actin*, elongation factor 1 alpha, *ef1α,* and ribosomal protein S18, *rps18*).

The analyses were performed in triplicate using 2.5 µL of iTaq Universal SYBR Green Supermix (Bio-Rad, El Prat de Llobregat, Spain), 250 nM of forward and reverse primers (Table 3) and 1 µL of diluted cDNA for each sample in a final volume of 5 µL. The reactions consisted of an initial denaturation step of 3 min at 95 °C, 40 cycles of 10 s at 95 °C, 30 s at 60 °C, followed by an amplicon dissociation analysis from 55 to 95 °C at 0.5 °C increase each 30 s [35]. Prior to the analyses, a dilution curve with a pool of samples was run to determine the appropriate cDNA dilution for each gene, as well as confirm the specificity of the reaction, and the absence of primer-dimers. The expression levels of each gene were calculated by the Pfaffl method [47], relative to the gene expression or geometric mean expression of the most stable reference gens analysed depending on the tissue (*ef1α* for pyloric caeca and *ef1α* and *rps18* for proximal intestine), using the Bio-Rad CFX Manager 3.1 software.

### 2.7. Statistical Analyses

The tanks were used as biological replicates in the analysis of biometric parameters [35], and the individual fish for the antioxidant enzyme activities and gene expression analyses. Prior to individual fish analysis, tank effect was checked for each parameter, but significant differences were not observed. All data were tested for normality by the Shapiro–Wilk test and homogeneity of variances by Levene’s test. If normality was met experimental values were compared using a one-way analysis of variance (ANOVA), and differences among means were tested for significance using a post hoc Tukey’s multiple range test. When the test for normality failed, the Kruskal–Wallis non-parametric test was used followed by Mann–Whitney U test. The significance level was set at *p* < 0.05. The software used for statistical analysis was SPSS (IBM-SPSS Statistics v.25.0, SPSS Inc., Chicago, IL, USA) and the one used for graphic representation was GraphPad 7.00 (GraphPad Software Inc. San Diego, CA, USA).

## 3. Results

### 3.1. Lipid Peroxidation

After 18 weeks of growth, levels of LPO (Table 4) were measured 24 h post feeding in pyloric caeca and proximal intestine of gilthead sea bream fed the experimental diets. In all experimental groups, LPO was higher in proximal intestine that in pyloric caeca; showing SLP group the LPO lowest levels in pyloric caeca and fish fed SLRP in proximal intestine.

Sea bream fed SL diet showed the highest levels of LPO in both intestinal regions. In this group, an increase in LPO by 98% in pyloric caeca and 71% in proximal intestine was found compared to fish fed S diet. However, the SL group presented a high individual variability, as it can be inferred from the high standard error of the mean. Thus, analyzing the results for this group more deeply, we found two very different subgroups of fish (Table 4). The first one (n = 6), called L-SL, presented a similar LPO amount and profile as the rest of the experimental treatments, with higher LPO levels in proximal intestine versus pyloric caeca. However, the second subgroup (n = 3, one fish per tank), called H-SL, showed towering levels of LPO in both intestinal regions compared to the rest of the groups. Since these differences suggested an unequal dietary adaptation inside this group of fish, the subgroups were considered from now on along the results section.

The addition of rapeseed or palm oils to the diets reduced LPO levels in both intestinal regions in comparison to SL-fed fish, being more evident in pyloric caeca (more than 95%) than in proximal intestine, where the reduction was associated mainly by the inclusion of palm in the diet (more than 75%) (Table 4). 

### 3.2. Antioxidant Enzyme Activity

**Figure 1:**
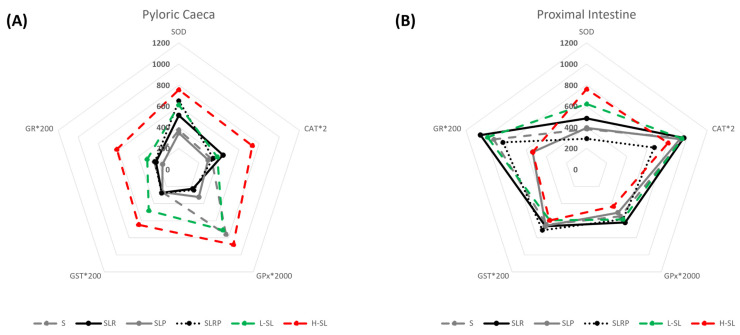
Antioxidant activities in pyloric caeca (**A**) and proximal intestine (**B**) at 24 h post feeding sea bream fed experimental diets. Enzyme activities are expressed as U *mg prot^−1^ and CAT, GPx, GST and GR were scaled to place them in the same chart and visualise better the differences between experimental conditions. Values are the mean (n = 3 in H-SL, n = 6 in S and L-SL, n = 9 in SLR, SLP and SLRP).

Pyloric caeca and proximal intestine antioxidant enzyme activities are shown in Table 5. Regarding SOD activity, no differences between intestinal segments were found, except for SLRP-fed fish that showed higher activity in pyloric caeca versus proximal intestine. Conversely, CAT, GST, and GR activities were higher in proximal intestine for all experimental conditions, although these differences disappeared in H-SL fish group where the enzyme activities showed a rise in pyloric caeca (Table 5). Finally, GPx activity presented a different regionalization pattern depending on the dietary treatment, being significantly higher in pyloric caeca from S and H-SL groups and in proximal intestine from SLR, SLP and SLRP fish (Table 5). Furthermore, when comparing the different enzymatic activities, we found that CAT activity was 10^2^ higher than GST and 10^3^ times higher than GPx at both intestinal regions (Table 5). These data on antioxidant activities were scaled and placed in a radial chart to better visualize the differences between dietary treatments (Figure 1). In pyloric caeca (Figure 1A, Table 5), the highest antioxidant activities were found in fish fed H-SL diet, followed by L-SL group, according to their unequal dietary adaptation. Instead, when incorporating palm oil to the blend of soybean and linseed oils (SLP group), the activity levels of SOD and CAT were significantly lower and different from the other experimental conditions, except for the S group (Figure 1A, Table 5). Conversely, the incorporation of rapeseed oil to the soybean and linseed oils blend induced CAT activity and showed intermediate levels of activity for SOD in pyloric caeca, while SLRP-fed fish presented the contrary, an increased SOD and intermediate CAT activities. Moreover, fish fed diets containing more than 50% of soybean oil (S and SL groups) presented significantly higher GPx activity than those fed with lower amounts of that oil (SLP, SLR, and SLRP), suggesting that soybean oil could trigger GPx activity (Figure 1A, Table 5). All groups showed similar GST activity (mean 1.35 ± 0.07 U × mg prot^−1^), except for H-SL and L-SL groups, that was higher. GR activity in pyloric caeca also presented the highest activity levels in H-SL fish (Figure 1A, Table 5).

In the proximal intestine, and similarly to what was found in the pyloric caeca, dietary linseed oil inclusion increased SOD activity by 62.5% in L-SL and 99.1% in H-SL fish versus fish fed S diet, whereas the inclusion of palm oil in the diet (SLP dietary group) downregulated the activity up to levels similar to those found in the S group (Figure 1B, Table 5). This significant downregulation of SOD activity was not seen in the SLR group. CAT activity of proximal intestine was similar for all experimental groups except SLRP, which showed the lowest values for CAT as well as SOD activities, and the highest values for GST. In this intestinal region, contrary to what happened in pyloric caeca, no changes were found in GPx activity between dietary treatments. Interestingly, in proximal intestine, a clear differential pattern in GR activity was detected when comparing L-SL and H-SL fish, being in the latter significantly lower than in the former (Figure 1B, Table 5). H-SL animals showed GR activities similar to those found in the SLP group, representing a decrease of the activity with respect to the rest of experimental conditions (Table 5).

### 3.3. Gene Expression

Gene expression of antioxidant enzymes at intestinal level 24 h post feeding was slightly modulated by the dietary treatments (Table 6). In this sense, in pyloric caeca, the expression of *sod2, gpx1,* and *gpx4* was upregulated in all dietary treatments compared to SLP group that showed the lowest values. Moreover, in proximal intestine only *gpx4* was modified showing the S group the highest value and the L-SL and the SLRP the lowest (Table 6).

### 3.4. Antioxidant Ratios

Since the activity of the antioxidant enzymes investigated can be affected by each other, either because the products of the activity of one enzyme are the reagents of the other or because they use the same substrate, the relationships between them were also calculated (Table 7). For all experimental groups, the (CAT+GPx)/SOD ratio was higher in the proximal intestine than in the pyloric caeca; in contrast, the ratio (GST+GPx/GR) was higher in the pyloric caeca, except for the H-SL group that showed similar activity ratios in both intestinal segments (Table 7). The dietary linseed oil inclusion caused a significant decrease in the (CAT+GPx)/SOD ratio in both intestinal segments of L-SL group versus diet S-fed fish; however, in H-SL animals, this decrease was only observed in proximal intestine. Moreover, in this intestinal segment, SLR animals showed a lower ratio than S, SLP and SLRP fish (Table 7). Regarding the CAT/GPx ratio, lower values were found in pyloric caeca from S and SL groups. Dietary palm oil inclusion (SLP group) contributed to increase this activity by 61%, whereas the SLR blend triggered a 254% increase in this ration in comparison to that observed in S, L-SL and H-SL fish. SLRP sea bream had intermediate values for this enzymatic ratio, between those observed for SLR and SLP animals, probably due to the lower palm oil content of this diet versus the SLP diet.

Instead, in proximal intestine, lower CAT/GPx ratios were found in sea bream fed diets containing rapeseed oil (SLR and SLRP) (Table 7). The highest levels in the (GST+GPx)/GR ratio for both intestinal segments were found in SLP-fed sea bream. In pyloric caeca, high levels of this ration were also measured in L-SL and S groups, being significantly lower for H-SL fish. In proximal intestine, the lowest values for the (GST+GPx)/GR ratio were observed in S and L-SL groups.

## 4. Discussion

ROS are generated by aerobic cellular metabolism in small quantities and most of the time, the intestine responds adequately against oxidative stress [48]. Nevertheless, external factors such as nutrition stress by both high-fat or high-carbohydrate diets could exacerbate the ROS production inducing tissue oxidative damage [20], negatively affecting intestinal functionality and health [48]. LPO is the hallmark of oxidative stress, and at intestine level is closely related with dietary lipid composition [49,50], since it depends on the amount of lipid and its length and unsaturation degree [19]. Accordingly, the inclusion of VO in fish feed formulation contributed to the reduction of intestinal LPO in comparison to sea bream fed FO diets [22]; on the contrary, plant protein diets contributed in sea bass to its increase in comparison with fish fed a fishmeal diet [51]. In the present study, LPO levels in SL group versus fish fed S diet were exacerbated, in accordance with previous results found by Magalhaes et al. [30] in sea bream fed diets supplemented with EPA and DHA, pointing to an inversely relationship of LPO levels with n-6/n-3 ratio. Nevertheless, this effect was not found in the rest of the groups fed diets including linseed oil, SLR, SLP and SLRP, which contained similar n-3, but lower levels of n-6, suggesting an effect on LPO of both the n-6/n-3 ratio and the n-6 content in the diet. In addition, the present results also showed an effect of dietary UFA/PUFA ratio on LPO in fish fed diets containing similar amounts of n-3 fatty acids. In this sense, fish fed the SLR diet, with a similar UFA/SFA ratio to that present in the SL-fed group, showed higher levels of LPO in pyloric caeca and proximal intestine than those found in SLP-fed sea bream with the lower UFA/SFA ratio, whereas fish fed SLRP diet showed globally intermediate LPO levels according to their halfway ratio.

Furthermore, the SL group of fish presented the highest LPO levels in both intestinal segments, but also showed high individual variability, since not all the fish fed this diet had high LPO levels, especially in the pyloric caeca region. This led to consider dividing this group into two subgroups with a different LPO profile and antioxidant activity in the pyloric region, which suggested an unequal adaptation to the administered diet. On one side, H-SL group presented exacerbated LPO levels and high antioxidant enzyme activities and on the other side, L-SL group, showed low LPO and moderate levels of SOD, CAT, and GR activities. Moreover, GST activity that is associated with a xenobiotic pathway was only regulated in the SL subgroup that presented the highest LPO, pointing to a higher reaction to toxics or allergens of the H-SL fish, mainly in pyloric caeca. In this regard, several studies showed a higher susceptibility to peroxidation of intestine versus liver due to its high turnover and dietary allergens or toxics exposure [52,53,54].

In addition, intestinal antioxidant enzyme activities also showed a modulation related to dietary lipid composition in an attempt to cope with the LPO generated to maintain a healthy oxidative status. In this sense, in the present study, an increase in SOD, CAT, GPx, and GR activities was found in pyloric caeca in the H-SL fish when compared with the other experimental groups and the L-SL fish. This suggests that this dietary VO blend triggers superoxide anion production in response to the high LPO generated in the H-SL group, stimulating SOD and in turn the enzymes involved in the second line of defence (CAT and GPx) as well as GR. Accordingly, antioxidant enzymes’ gene expression was generally highest in the H-SL group. On the other hand, pyloric caeca from SLP fish showed the lowest peroxidation levels, in agreement with the low gene expression and activity of antioxidant enzymes. The differences in the profile of antioxidants’ activities found between intestinal segments in SLRP-fed sea bream versus the other experimental groups could also be related to the different LPO levels found, which were higher in pyloric caeca than in proximal intestine. In this sense, low SOD and CAT activities were detected in proximal intestine from SLRP-fed sea bream, whereas their activities were high and intermediate in pyloric caeca.

As previously mentioned, the mixture of soybean and linseed oils (SL diet) is not the most appropriate for sea bream since LPO can be triggered, which did not occur when palm and/or rapeseed oils were included in the mixture of VO. Additionally, the dietary fatty acids profile affects, in addition to LPO levels, the activity of antioxidant enzymes. Specifically, SOD activity, the first line of defence against oxidative stress, was highly modulated by the dietary lipid profile, with the S, SL, SLP, and SLR groups showing a similar change in pattern and magnitude of activity in both intestinal segments. In this sense, dietary inclusion of rapeseed oil and/or especially palm oil seems to promote the reduction of SOD activity whose increase could be related with the dietary addition of linseed oil. CAT and GPx constitute the second line of defence, removing H_2_O_2_ produced by SOD [20]. CAT acts in high oxidative stress conditions [20,55] and its activity was low only in pyloric caeca of fish fed diets with palm oil inclusion (SLP and SLRP) and in proximal intestine in SLRP-fed sea bream. Instead, GPx copes with oxidative stress in basal conditions [20,55]. Regarding this antioxidant enzyme, low levels of activity were found in pyloric caeca from sea bream fed with the SLP diet and especially in those fed with SLR and SLRP diets, which were different from those found in S and SL sea bream. These results pointed to a downregulation in GPx activity by the presence of rapeseed oil and an upregulation by soybean oil levels above 50% in pyloric caeca, whereas no modulation was found in proximal intestine.

Among the studies conducted at intestinal level on oxidative stress, this is the first, to our knowledge, in which the pyloric caeca and proximal intestine are analysed at the same time. The present results showed that LPO was higher in proximal intestine than in pyloric caeca, except in SL fish, and antioxidant activities levels were also different between these intestinal regions. Thus, the anatomical location of pyloric caeca, anterior to proximal intestine, and their specific functions could in part explain the differences found. Pancreatic and bile juices are released in pyloric caeca and in then alkaline digestion starts [56]. Moreover, this region is subjected to a higher enterocyte renewal rate than proximal intestine [57] and presents a characteristic mixing and retrograde contractile activity [58]. These characteristics contribute to slowing down the intestinal transit favouring the chyme to be retained in the pyloric caeca for a short time until it gradually passes into the proximal intestine [59,60], where digestion will finish and nutrient absorption will take place. An intestinal regionalization has been previously described in studies related to digestive enzyme activities, diet, and temperature [61,62,63,64,65,66,67], showing how pyloric caeca and proximal intestine functions were differently modulated. In the present study, a clear dietary modulation of most of the antioxidant enzymes studied in pyloric caeca and only SOD in proximal intestine was observed. This idea was also reinforced by the low gene expression changes found. Moreover, in proximal intestine, the antioxidant capacity of CAT, GPx, GST, and GSH recycling by GR were generally higher than in pyloric caeca, regardless of the diet administered. Thus, data suggest the attempt to preserve the epithelium of proximal intestine, which has a lower rate of cell renewal, mature enterocytes [57,68] and has greater digestive and absorptive capacities than those found in the pyloric caeca region [56]—even going so far as to affect the proper function of the region of the pyloric caeca in the case of H-SL animals. Thus, this group reached very high levels of LPO in pyloric caeca that would lead to a higher cellular renewal rate, which would prevent proper cell maturation for the performance of digestive and absorptive functions, possibly aiming to maintain as low as possible peroxidation levels in the proximal intestine. This idea agrees with other studies where the effects of antinutritional factors and enteritis in Atlantic salmon were analysed [69,70,71]. In fact, antioxidant ratios calculated in the present study also support this possible regulation since the (CAT+GPx)/SOD ratio was higher in proximal intestine; whereas, the (GST+GPx)/GR ratio, a pathway related with xenobiotics and GSH reduction, was higher in PC, being both processes directed to maintaining the functionality of proximal intestine.

GR is involved in GSSH recycling to GSH. The latter is a non-enzymatic antioxidant that directly quenches ROS or indirectly acts as a substrate for both GPx and GST [20], being a mechanism extensively used by the intestine due to their susceptibility to oxidation and the turnover rate of enterocytes [53]. In this context, in H-SL fish, higher GR activity was found in pyloric caeca than in proximal intestine according to the high LPO levels that were accompanied by a higher activity of GPx and GST. In this group, the difference in GR activity between both segments could be due to a different use of GSH to remove ROS. Instead, the low GR activity found in fish fed the SLP diet may be due to the low LPO levels found in both pyloric caeca and proximal intestine.

Considering the possible benefits and disadvantages of the diets, it is well known that fish fed a diet containing only soybean oil present lipid accumulation in enterocytes that could led to an impairment in the digestion process [6,15,16,17]. Our results showed that the SL diet also had also negative effects in the gut since it induced, in some animals, exacerbated levels of LPO in both intestinal regions; revealing their inability to adapt to the diet. Moreover, fish fed with the SLR diet presented higher oxidative damage in proximal intestine versus SLP and SLRP fed sea bream. Since the proximal intestine area is principally involved in the processes of completion of digestion and nutrient absorption, the SLR diet would not be the most recommended for this species. Regarding the last two groups, SLP and SLRP, the main differences were that in the latter, the highest oxidative damage was found in pyloric caeca, a region involved in lipid absorption [56], and presented an increase mainly in SOD activity which would lead to a greater energy expenditure to cope with oxidative stress. On the contrary, the SLP diet would be the optimal one considering the intestinal redox status, since SLP-fed fish presented low and moderate activities in all the antioxidants enzymes studied, but that allowed them to also keep low LPO levels, therefore suggesting a lower energy expenditure need to cope with dietary induced LPO at intestinal level.

## 5. Conclusions

In summary, this study shows for the first time the interest of the pyloric caeca region in coping with the oxidative stress generated by the inclusion of VO in fish diets. Moreover, it also highlights the importance of the proportion of different VO in these mixtures, being in this case the inclusion of linseed oil a factor to consider in dietary formulation since it can negatively affect the oxidative status of the animal at intestinal level. To conclude, among all the diets evaluated, the SLP diet induced in sea bream the best intestinal oxidative status, with low LPO levels and antioxidant enzyme activities.

## Figures and Tables

**Table 1:** Oil composition and Vitamin/Mineral (Vit/Min) premix and proximate composition of the experimental diets.

	**Diets**				
**Ingredients (%)**	**S**	**SL**	**SLR**	**SLP**	**SLRP**
Fish oil	4.64	4.64	4.64	4.64	4.64
Palm oil	-	-	-	4.81	2.43
Linseed oil	-	2.82	2.42	3.37	2.7
Rapeseed oil	-	-	5.80	-	5.84
Soybean oil	13.63	10.81	5.39	5.54	2.65
Vit/Min premix	1.78	1.78	1.8	1.69	1.79
	**Proximate composition (%)**				
Dry matter	93.21	93.21	93.21	93.21	93.21
Crude protein	46.5	46.5	46.50	46.45	46.33
Crude fat	22.4	21.9	22.40	22.40	21.90
Ash	5.7	5.7	5.69	5.66	5.66

S: diet containing only soybean oil; SL: diet containing soybean and linseed oil; SLR: diet containing soybean, linseed and rapeseed oil; SLP: diet containing soybean, linseed and palm oil; SLRP: diet containing soybean, linseed, rapeseed and palm oils.

**Table 2:** Dietary fatty acid profile of the experimental diets.

	Diets				
Fatty Acid (%)	S	SL	SLR	SLP	SLRP
C14:0	2.15	2.12	2.14	2.33	2.27
C16:0	13.27	12.55	10.95	18.98	14.17
C16:1n-7	2.34	2.31	2.37	2.36	2.40
C16:2n-6	0.29	0.28	0.30	0.28	0.29
C18:0	2.87	3.16	2.80	3.51	2.99
C18:1n-9	19.88	19.19	27.30	23.02	29.81
C18:1n-7	2.03	1.88	2.24	1.62	2.15
C18:2n-6	36.51	31.95	24.93	21.98	19.10
C18:3n-3	4.37	10.11	10.11	9.83	10.04
C18:4n-3	0.75	0.73	0.73	0.70	0.74
C20:1 sum. isomers	1.80	1.85	2.15	1.70	2.05
C20:4n-6	0.29	0.23	0.22	0.23	0.22
C20:4n-3	0.22	0.23	0.22	0.25	0.24
C20:5n-3 EPA	3.02	3.02	3.10	3.02	3.15
C22:1 sum. isomers	2.13	2.06	2.24	2.18	2.17
C22:5n-3	0.44	0.43	0.46	0.43	0.46
C22:6n-3 DHA	2.98	2.87	2.99	2.95	2.95
C24:1n-9	0.25	0.23	0.27	0.27	0.26
SFA not listed	1.06	0.97	1.01	0.87	0.96
Monoenes not listed	0.10	0.14	0.12	0.10	0.11
n-6 FA not listed	0.20	0.24	0.25	0.21	0.22
n-3 FA not listed	0.22	0.20	0.20	0.17	0.20
Others	0.36	0.36	0.32	0.32	0.36
Sum. SFA	19.35	18.80	16.90	25.69	20.39
Sum. MUFA	28.53	27.66	36.69	31.25	38.95
Sum. n-6 FA	37.29	32.70	25.70	22.70	19.83
Sum. n-3 FA	12.0	17.59	17.81	17.35	17.78
UFA/SFA	4.02	4.15	4.75	2.78	3.75
MUFA/PUFA	0.58	0.55	0.84	0.78	1.04
n-6/n-3	3.11	1.86	1.44	1.31	1.12
Unknown	2.50	2.90	2.60	2.70	2.70

EPA: Eicosapentaenoic acid; DHA: Docosahexaenoic acid; FA: Fatty acids; SFA: Saturated fatty acids; MUFA: Monounsaturated fatty acids; MUFA/PUFA: Monounsaturated fatty acids/Polyunsaturated fatty acids; UFA/SFA: Unsaturated fatty acids/Saturated fatty acids.

**Table 3:** Primers used for real-time qPCR: gene name, sequence, annealing temperature (Ta) and GenBank accession numbers.

Gene		Sequence (5′-3′)	Tm (°C)	Accession Number
*β-actin*	F R	TCCTGCGGAATCCATGAGA GACGTCGCACTTCATGATGCT	60	X89920
*ef1α*	F R	CTTCAACGCTCAGGTCATCAT GCACAGCGAAACGACCAAGGGGA	60	AF184170
*rps18*	F R	GGGTGTTGGCAGACGTTAC CTTCTGCCTGTTGAGGAACCA	60	AM490061.1
*cat*	F R	TTCCCGTCCTTCCATTCACTC CTCCAGAAGTCCCACACCAT	60	FG264808
*gpx1*	F R	GAAGGTGGATGTGAATGGAAAAGATG CTGACGGGACTCCAAATGATGG	60	DQ524992
*gpx4*	F R	TGCGTCTGATAGGGTCCACTGTC GTCTGCCAGTCCTCTGTCGG	60	AM977818
*gr*	F R	CAAAGCGCAGTGTGATTGTGG CCACTCCGGAGTTTTGCATTTC	60	AJ937873
*gst3*	F R	CCAGATGATCAGTACGTGAAGACCGTC TGCTGATGTGAGGAATGTACCGTAAC	60	JQ308828
*sod1*	F R	CCATGGTAAGAATCATGGCGG CGTGGATCACCATGGTTCTG	60	AJ937872
*sod2*	F R	CCTGACCTGACCTACGACTATGG AGTGCCTCCTGATATTTCTCCTCTG	60	JQ308832

*β-actin*: beta actin; ef1α: elongation factor 1 alpha; *rps18*: ribosomal protein S18; *cat*: catalase, *gpx1*: mitochondrial glutathione peroxidase 1; *gpx4*; cytosolic glutathione peroxidase, *gr*: glutathione reductase; *gst*: glutathione-S-transferase; *sod1*: superoxide dismutase 1 and *sod2*: superoxide dismutase 2. F: forward, R: reverse.

**Table 4:** Lipid peroxidation levels (nMols MDA × mg prot^−1^) in pyloric caeca and proximal intestine 24 h post feeding in sea bream fed the experimental diets.

Dietary Conditions	Pyloric Caeca	Proximal Intestine
S	0.057 ± 0.006 ^c^	0.318 ± 0.039 ^mn^*
SL	2.907 ± 1.109	1.097 ± 0.502
L-SL	0.107 ± 0.006 ^b^	0.201 ± 0.015 ^n^*
H-SL	9.440 ± 2.750 ^a^*	2.889.2 ± 1.407 ^m^
SLR	0.139 ± 0.017 ^b^	0.643 ± 0.122 ^m^*
SLP	0.038 ± 0.005 ^d^	0.265 ± 0.019 ^no^*
SLRP	0.136 ± 0.022 ^b^	0.227 ± 0.011 ^o^

Values are the mean ± SEM (n = 6 in S, n = 9 in SL, SLR, SLP and SLRP). SL group was subdivided into two subgroups according to LPO amount, L-SL (n = 6) and H-SL (n = 3). Significant differences between dietary conditions are shown by different letters, from a to d in pyloric caeca, and from m to o in proximal intestine (*p* < 0.05). Differences between intestinal segments within the same condition are shown by an asterisk at the highest value (*p* < 0.05). SL group (grey background) was excluded from the statistical analyses.

**Table 5:** Antioxidant enzymes activities (U × mg prot^−1^) in pyloric caeca and proximal intestine 24 h post-feeding in sea bream fed the experimental diets.

**Pyloric Caeca**					
**Dietary Conditions**	**SOD**	**CAT**	**GPx**	**GST**	**GR**
S	374.6 ± 35.0 ^c^	163.0 ± 11.3 ^bc^	0.380 ± 0.111 ^ab^*	1.33 ± 0.11 ^b^	1.22 ± 0.10 ^b^
L-SL	611.6 ± 93.9 ^ab^	191.9 ± 9.0 ^ab^	0.355 ± 0.043 ^a^	2.41 ± 0.22 ^a^	1.57 ± 0.17 ^bc^
H-SL	754.4 ± 173 ^a^	365.8 ± 69.4 ^a^	0.440 ± 0.060 ^a^*	3.23 ± 0.57 ^a^	3.08 ± 0.34 ^a^
SLR	513.2 ± 41.8 ^b^	220.3 ± 4.4 ^a^	0.113 ± 0.009 ^c^	1.36 ± 0.06 ^b^	1.13 ± 0.09 ^b^
SLP	345.8 ± 43.3 ^c^	145.5 ± 6.2 ^c^	0.161 ± 0.012 ^b^	1.33 ± 0.06 ^b^	0.80 ± 0.07 ^c^
SLRP	650.5 ± 44.3 ^ab^*	171.6 ± 9.1 ^b^	0.121 ± 0.008 ^c^	1.38 ± 0.06 ^b^	1.19 ± 0.11 ^b^
**Proximal intestine**					
**Dietary conditions**	**SOD**	**CAT**	**GPx**	**GST**	**GR**
S	381.9 ± 33.4 ^op^	479.5 ± 16.3 ^m^*	0.274 ± 0.030	3.23 ± 0.22 ^mn^*	4.62 ± 0.45 ^m^*
L-SL	620.7 ± 72.8 ^m^	474.5 ± 42.5 ^m^*	0.292 ± 0.035	2.97 ± 0.12 ^n^	4.93 ± 0.46 ^m^*
H-SL	760.5 ± 151.5 ^m^	405.3 ± 18.9 ^m^	0.216 ± 0.019	2.99 ± 0.17 ^mn^	2.68 ± 0.26 ^n^
SLR	483.7 ± 56.62 ^mn^	483.5 ± 32.2 ^m^*	0.310 ± 0.030 *	3.32 ± 0.19 ^mn^*	5.28 ± 0.66 ^m^*
SLP	394.4 ± 40.24 ^no^	464.4 ± 27.3 ^m^*	0.252 ± 0.018 *	3.30 ± 0.15 ^mn^*	2.68 ± 0.12 ^n^*
SLRP	292.0 ± 29.53 ^p^	336.7 ± 10.5 ^n^*	0.292 ± 0.024 *	3.55 ± 0.20 ^m^*	4.16 ± 0.29 ^m^*

Values are the mean ± SEM (n = 3 in H-SL, n = 6 in S and L-SL, n = 9 in SLR, SLP and SLRP). Significant differences between dietary conditions for pyloric caeca (grey background) or proximal intestine (white background) are shown by different letters, from a to c in pyloric caeca and from m to o in proximal intestine (*p* < 0.05). Differences between intestinal segments within the same condition are shown by an asterisk at the highest value (*p* < 0.05).

**Table 6:** Relative gene expression of antioxidant enzymes in pyloric caeca and proximal intestine 24 h post-feeding in sea bream fed the experimental diets.

	**Pyloric Caeca**						
**Dietary** **Conditions**	***sod1***	***sod2***	***cat***	***gpx1***	***gpx4***	***gst***	***gr***
S	2.15 ± 0.52	3.09 ± 1.00 ^a^	2.42 ± 0.74	1.91 ± 0.38 ^a^	3.32 ± 0.94 ^a^	1.70 ± 0.47	1.35 ± 0.43
L-SL	1.60 ± 0.49	2.05 ± 0.53 ^a^	1.47 ± 0.29	1.01 ± 0.22 ^b^	3.24 ± 2.05 ^ab^	0.89 ± 0.25	1.51 ± 0.74
H-SL	2.30 ± 1.08	3.60 ± 1.76 ^a^	3.13 ± 1.79	2.19 ± 0.98 ^ab^	4.05 ± 2.85 ^ab^	1.88 ± 0.74	1.98 ± 1.05
SLR	1.63 ± 0.39	1.79 ± 0.53 ^ab^	1.63 ± 0.27	1.43 ± 0.19 ^ab^	1.26 ± 0.30 ^bc^	1.51 ± 0.45	1.17 ± 0.25
SLP	0.57 ± 0.17	0.55 ± 0.17 ^b^	0.67 ± 0.22	0.37 ± 0.11 ^c^	0.45 ± 0.14 ^c^	0.63 ± 0.22	0.51 ± 0.20
SLRP	1.65 ± 0.27	1.55 ± 0.21 ^a^	1.26 ± 0.17	1.58 ± 0.32 ^ab^	1.29 ± 0.20 ^ab^	1.45 ± 0.28	1.36 ± 0.19
**Proximal intestine**							
**Dietary ** **conditions**	***sod1***	***sod2***	***cat***	***gpx1***	***gpx4***	***gst***	***gr***
S	0.87 ± 0.09	1.72 ± 0.19	0.87 ± 0.21	2.04 ± 0.20	0.92 ± 0.12 ^m^	1.04 ± 0.08	1.73 ± 0.19
L-SL	0.87 ± 0.20	1.87 ± 0.37	0.86 ± 0.09	1.79 ± 0.14	0.55 ± 0.04 ^o^	0.90 ± 0.30	1.95 ± 0.42
H-SL	0.69 ± 0.14	2.80 ± 1.21	1.01 ± 0.28	2.97 ± 1.81	0.65 ± 0.16 ^mno^	0.91 ± 0.14	3.54 ± 1.83
SLR	0.85 ± 0.10	1.80 ± 0.22	1.20 ± 0.18	1.93 ± 0.09	0.87 ± 0.06 ^m^	1.24 ± 0.25	2.17 ± 0.26
SLP	0.75 ± 0.21	1.60 ± 0.15	0.82 ± 0.15	1.69 ± 0.20	0.73 ± 0.06 ^mn^	0.97 ± 0.10	1.80 ± 0.25
SLRP	0.75 ± 0.11	1.44 ± 0.20	0.72 ± 0.11	1.80 ± 0.21	0.55 ± 0.08 ^no^	0.88 ± 0.16	1.79 ± 0.14

Values are the mean ± SEM (n = 3 in H-SL, n = 6 in S and L-SL, n = 9 in SLR, SLP and SLRP). Significant differences between dietary conditions for pyloric caeca (grey background) or proximal intestine (white background) are shown by different letters, from a to c in pyloric caeca and from m to o in proximal intestine (*p* < 0.05).

**Table 7:** Ratios of antioxidant enzymes activities (CAT+GPx)/SOD, CAT/GPx and (GST+GPx)/GR ratios in pyloric caeca and proximal intestine 24 h post feeding in sea bream fed the experimental diets.

**Pyloric Caeca**			
**Dietary Conditions**	**(CAT+GPx)/SOD**	**CAT/GPx**	**(GST+GPx)/GR**
S	0.468 ± 0.036 ^a^	569.8 ± 95.1 ^e^	1.566 ± 0.126 ^ab^*
L-SL	0.272 ± 0.017 ^b^	598.0 ± 40.0 ^e^	1.924 ± 0.21 ^a^*
H-SL	0.497 ± 0.041 ^a^	771.9 ± 47.5 ^d^	1.082 ± 0.116 ^c^
SLR	0.480 ± 0.047 ^a^	2289.3 ± 215.8 ^a^*	1.326 ± 0.101 ^bc^*
SLP	0.580 ± 0.096 ^a^	1043.5 ± 98.4 ^c^	1.982 ± 0.142 ^a^*
SLRP	0.311 ± 0.029 ^b^	1488.8 ± 67.0 ^b^	1.378 ± 0.166 ^abc^
**Proximal intestine**			
**Dietary conditions**	**(CAT+GPx)/SOD**	**CAT/GPx**	**(GST+GPx)/GR**
S	1.388 ± 0.098 ^m^*	1997.4 ± 218.1 ^mn^*	0.693 ± 0.014 ^o^
L-SL	0.645 ± 0.023 ^o^*	1781.5 ± 303.5 ^mn^*	0.576 ± 0.015 ^o^
H-SL	0.730 ± 0.150 ^no^	2008.2 ± 240.1 ^mn^*	1.226 ± 0.053 ^m^
SLR	0.916 ± 0.038 ^n^*	1569.8 ± 148.7 ^n^	0.914 ± 0.090 ^n^
SLP	1.443 ± 0.121 ^m^*	1956.1 ± 125.4 ^m^*	1.399 ± 0.022 ^m^
SLRP	1.351 ± 0.115 ^m^*	1432.9 ± 101.3 ^n^	1.095 ± 0.023 ^n^

Values are the mean ± SEM (n = 3 in H-SL, n = 6 in S and L-SL, n = 9 in SLR, SLP and SLRP). Significant differences between dietary conditions for pyloric caeca (grey background) or proximal intestine (white background) are shown by different letters, from a to e in pyloric caeca, and from m to o in proximal intestine (*p* < 0.05). Differences between intestinal segments within the same condition are shown by an asterisk at the highest value (*p* < 0.05).

## Data Availability

Not applicable.
